# Adaption to Extreme Rainfall with Open Urban Drainage System: An Integrated Hydrological Cost-Benefit Analysis

**DOI:** 10.1007/s00267-012-0010-8

**Published:** 2013-01-19

**Authors:** Qianqian Zhou, Toke Emil Panduro, Bo Jellesmark Thorsen, Karsten Arnbjerg-Nielsen

**Affiliations:** 1 Department of Environmental Engineering, Technical University of Denmark, Miljoevej, Bldg 113 DK-2800 Kgs, 2800 Lyngby, Denmark; 2 Department of Food and Resource Economics, Faculty of Science, University of Copenhagen, Frederiksberg C, Denmark; 3 Department of Food and Resource Economics and Centre of Macroecology, Evolution and Climate, Faculty of Science, University of Copenhagen, Frederiksberg C, Denmark

**Keywords:** Hedonic valuation, Urban green areas, Water, Urban drainage, Climate change

## Abstract

This paper presents a cross-disciplinary framework for assessment of climate change adaptation to increased precipitation extremes considering pluvial flood risk as well as additional environmental services provided by some of the adaptation options. The ability of adaptation alternatives to cope with extreme rainfalls is evaluated using a quantitative flood risk approach based on urban inundation modeling and socio-economic analysis of corresponding costs and benefits. A hedonic valuation model is applied to capture the local economic gains or losses from more water bodies in green areas. The framework was applied to the northern part of the city of Aarhus, Denmark. We investigated four adaptation strategies that encompassed laissez-faire, larger sewer pipes, local infiltration units, and open drainage system in the urban green structure. We found that when taking into account environmental amenity effects, an integration of open drainage basins in urban recreational areas is likely the best adaptation strategy, followed by pipe enlargement and local infiltration strategies. All three were improvements compared to the fourth strategy of no measures taken.

## Introduction

While climate change predictions are inherently uncertain, the predictions of future changes in precipitation patterns seem fairly robust for Northern Europe (van der Linden and Mitchell 2009). The anticipated climate change will affect and increase precipitation extremes, leading to an increase in design intensities of at least 20 % (Madsen and others 2009; Arnbjerg-Nielsen 2012). This poses a challenge to urban drainage design as future drainage systems will have to deal with increased frequency and volume of storm water flows. As a result, the urban drainage capacity needs to be significantly increased in many parts of Northern Europe, including the case area in Denmark addressed in this study (Arnbjerg-Nielsen and Fleischer 2009). There are, however, increased concerns that expanding the underground pipe system is not a sustainable solution for climate adaptation in the long term or that attractive alternatives exist (Roy and others 2008; Zevenbergen and others 2008; Wong and Eadie 2000).

There is increasing acknowledgment of the potentials of decentralized drainage system based on local treatment, attenuation, re-use, retention, and infiltration of precipitation runoffs (Ashley and others 2007; Roy and others 2008; Stahre 2006). Depending on design, such decentralized solutions may promote a more sustainable development by adding also to esthetics, social, and environmental values in the urban area. In many respects, a decentralized system can substitute or be integrated into the conventional sewer system. If carefully planned, a decentralized system can be a part of the green infrastructure in urban area, thus meeting demands for both climate change adaptation and urban recreational services.

The idea of decentralized drainage system has been promoted through, and as part of, the idea of local community activism for climate change adaptation. The focus has been on small-scale systems in which local property owners could implement on their own properties, typically by means of underground infiltration units. We will denote these systems, local urban drainage systems (LUDS). A common characteristic of LUDS is that they do not impact on the urban landscape in ways that provide additional recreational benefits. In general, LUDS will go unnoticed to the public eye. LUDS must develop into large-scale systems to have an impact on amenity value. As an alternative strategy to green roofs, water trenches, and rain gardens, one could consider transforming the urban landscape, e.g., by creating small lakes and green spaces. Appropriately designed such large-scale open urban drainage systems could both serve as places of recreational experience and as a significant temporary rainwater storage capacity during extreme rain events. We will name these large-scale systems, open urban drainage systems (OUDS), as they are open to the air and to the general public and may provide a range of recreational services, which the small-scale LUDS do not.

The implementation of LUDS and OUDS is not straightforward. Decision-makers need tools to react to the challenges ahead in an economically rational manner. There have been many visionary demonstrations of the decentralized solutions but only a few have come up with appropriate technical and economic tools to underpin their efficiency (Marsalek and Chocat 2002; Stahre 2006; Wong and Eadie 2000). More efforts are needed to further study their effects on extreme events as well as the costs and benefits (Ashley and others 2007; Hellström and others 2000; Wong and Eadie 2000). Risk-based economic assessment is a fundamental method for climate adaptation assessment; however, the majority of such economic analyses remain in the form of traditional budget cost-benefit analysis (CBA), see, e.g., Gafni (2006), which only accounts for the impacts in a hydrological context. In our study, the expansion of possible approaches to urban storm water management caused us to extend the CBA to include estimates of the welfare economic measures of non-market effects in the form of recreational effects from the proposed OUDS.

We evaluate the performance of four distinct strategies to handle the expected changes in extreme rainfall events. The first is a baseline strategy, *the laissez*-*faire strategy*, which assumes that urban storm water is to be handled by existing infrastructure only. The second strategy, *the business*-*as*-*usual (BAU)* (Baura 2006) *strategy*, assumes that increased drainage capacity is obtained by means of expansion of sewer pipes and concrete rainwater basins when necessary.^1^ The third strategy, *the infiltration strategy*, builds on a LUDS approach where property owners implement rainwater trenches in their gardens. The LUDS will infiltrate rainwater on a day-to-day basis and will serve as a temporal storage capacity during larger rainfall events. The fourth strategy is the *OUDS*, which exploit the existing green spaces and implement lakes which will temporally allow for massive influx of rainwater during a rain event. In short, such OUDS solutions essentially are rainwater basins integrated in pleasant green areas, which provide additional recreational benefits within the urban landscape. The value of the additional recreational amenities from the potential OUDS is estimated using hedonic house price valuation capturing the value of the surrounding neighborhood. When implementing this strategy in our case study area, it is necessary to convert some private properties into green spaces to provide room for OUDS. This implies additional costs for obtaining the benefits.

To evaluate the performance of the four strategies, we established a cross-disciplinary model, which integrated techniques of risk assessment with flood inundation modeling, climate change, environmental evaluation tools, and socio-economic tools to uncover the costs and benefits associated with the strategies. A budget-oriented CBA approach is insufficient as a decision-maker tool as it will be blind to the potential additional non-market services (negative and positive) provided by the urban water infrastructure.

## Methods

**Fig. 1:**
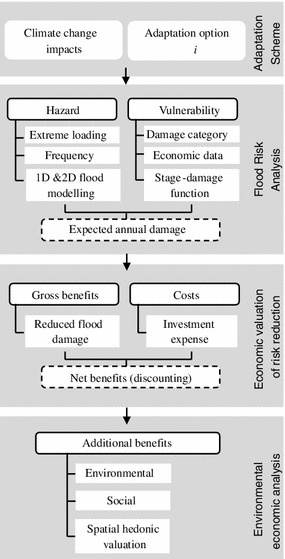
The stepwise procedure of the cross-disciplinary framework for evaluating the alternative adaptation strategies

The general procedure of the cross-disciplinary framework is shown in Fig. 1. It contains a comprehensive urban inundation model and several detailed economic models. The adaptation scheme describes the anticipated climate change impacts in an area as well as the planned adaptation alternatives. The flood risk analysis is performed on the basis of a flood risk assessment framework estimating both hazard and vulnerability characteristics of the area under the investigated adaptation strategy. Economic valuation of risk reduction is assessed using a step-by-step approach to aggregate the gross benefits and costs of the adaptation strategy in the context of risk reduction. The methodological background of the flood risk analysis and the step-by-step approach is a coherent economic pluvial flood risk assessment framework for evaluation of climate change adaptation options in a hydrological context developed by Zhou and others (2012). Finally, the environmental economic analysis applies a hedonic valuation approach to capture at least a substantial part of the value of externalities related to the urban water infrastructure.

### Current State and Development of Urban Infrastructure and City Planning

With increasing recognition of climate impacts on urban flood risk, there is a strong need to adapt urban infrastructure to reduce the substantial economic losses from extreme climatic events. While planning a climate change adaptation scheme, in general, several infrastructure development scenarios need to be constructed and assessed. A comparative cost-benefit assessment is often necessary to provide decision-makers with a firm basis for selecting the appropriate adaptable solution. Therefore, each scenario will be analyzed through the cross-disciplinary framework to compare their performance in terms of costs and benefits.

### Flood Risk Analysis and Integration in CBA

Flood risk analysis is the fundamental procedure for climate adaptation assessment. To assess the risk level of flooding in an area, an analysis of hazards and vulnerabilities is required. Hazards describe the extreme climatic loadings, such as a range of occurrence probabilities for different flood events and the extent and depth of these floods. In general, each occurrence probability is described by the equivalent return period, which is a statistic measure of the average recurrence interval of an extreme climatic loading (Haynes and others 2008). Vulnerabilities describe the spatial distribution of susceptible groups and properties to flooding and the potential adverse effects caused by exposure of these vulnerabilities to the hazards, e.g., the number of houses flooded, or the number of people exposed for a given loading.

The flood risk posed by extreme rain events was simulated using a comprehensive 1D–2D coupled urban inundation model. Such a model can simulate one-dimensional pipe flow underground and two-dimensional surface flow patterns. The pipe flow is simulated by the 1D sewer model and the surface flow is simulated by the 2D overland flow model. There are a number of connections between the two models (e.g., manholes, open channels) allowing water exchange dynamically (Domingo and others 2010; Mark and others 2004; Mike By DHI 2011). Runoff from build-up areas due to precipitation is first collected through subcatchments and generated in the 1D sewer model. As flow increases, water can flow out to the surface through the connections. Depending on the flow conditions, water can also flow back to the sewer system in the modeling process. Input data in the simulation include a description of the rainfall, models of the drainage system, a digital elevation model (DEM), and parameter descriptions for water exchange between the 1D and 2D simulations. The resulting outcomes are a range of flood hazard maps that show the locations of inundation and the simulated maximum water depths for a range of return periods covering the time period during which the strategies are evaluated.

In the vulnerability analysis, mainly physical impacts were investigated, such as damage to houses, basements, and roads. Some intangible losses were taken into account, including traffic delay, pollution of recreational sites, and health impacts. With a spatial distribution of the land use and socio-economic data of an area, we used a “threshold principle” to identify the affected damage categories in a GIS-based risk model based on the simulated inundation depth maps from the hazard analysis. Such a threshold principle adopts a binary approach: “flooded or not flooded” due to the lack of sufficient information on the staged-depth-damage function (Kubal and others 2009; Zhou and others 2012). As a result, the damage was identified as a result of exposure of vulnerable properties to the hazards and was modeled depending only on whether the inundation depth exceeds the threshold or not. The threshold level differs between damage categories and uniform unit costs are assigned to the flooded units when water depth rises above their critical thresholds. Further details on damage categories, threshold levels, and costs are provided in Zhou and Arnbjerg-Nielsen (submitted). Finally, the damage costs were estimated for different flood events by multiplying the affected units by the corresponding unit costs, respectively. The final outcome was expressed in terms of expected annual damage (EAD) as a measure of flood risk level of an area.

The flood risk analysis and damage assessment were integrated into a CBA, assessing the performance of each alternative adaptation strategy in the form of net present value, using a discount rate of 3 % (Pearce and others 2006). We adjusted the actual design of each adaptation strategy in the case area in a heuristic manner to maximize the resulting cost-benefit measure of each. The costs in the CBA included the investment expenses of a planned adaptation in this study, e.g., infrastructure establishments, and the gross benefits were calculated as saved damage costs by means of EADs from the risk assessment to account for the flood frequency and damage estimation.

### Environmental Economic Analysis: Hedonic House Price Valuation

We used the hedonic house price valuation method to estimate the marginal willingness to pay for proximity to urban green spaces of various types. Previous studies on hedonic house price valuation have found that amenity services provided by green spaces have clear impacts on property prices in nearby residential areas. Attributes such as tree cover, maintenance, and management have been found to have distinct property price signals, which reflects the underlying preference for the different attributes within the same general environmental good (Anthon and others 2005; Bark and others 2009; Jiao and Liu 2010; Mansfield and others 2005).

Urban green spaces are not a uniform amenity. Accessibility, size, and the presence of a lake and/or tree cover provide different recreational opportunities within the urban green spaces. In the hedonic valuation analysis here, we distinguish between these categories as found empirically relevant, cf. below.

#### The Theoretical Basis of the Hedonic Valuation Method

The theoretical foundation of the hedonic valuation method was developed, among others and in particular, by Rosen (1974), and further developed by e.g., Palmquist (1992, 2005). We refer the reader to these and other references for the details, but here it suffices to explain that the basic idea of the method is that in equilibrium, the price *P* of any given house, *n*, can be modeled as a function of a vector *z* that includes all *K* house characteristics, *z*_*ik*_. The hedonic price function may be formulated as follows:

1 $$ P_{n} = f\left( {z_{n1} , \ldots ,z_{nk} , \ldots z_{nK} ;\Uptheta } \right), $$

where Θ is a set of parameters related to the characteristics and is specific to the housing market considered. Note that the characteristics may also include environmental attributes and values obtained by ownership of the house, in this context proximity and access to urban green areas. Assuming weak separability with respect to the parameters of interest insures that the marginal rate of substitution between any two characteristics is independent of the level of all other characteristics. With that assumption in place, the implicit price of a house characteristic *z*_*nk*_ is a measure of the Marginal Willingness To Pay, $MWTP = {{dP_{n} } \mathord{\left/ {\vphantom {{dP_{n} } {z_{nk} }}} \right. \kern-0pt} {z_{nk} }}$ for this house characteristic (Palmquist 1992). This allows us to estimate the value of a small change in the environmental good.

The hedonic price function only provides information on one point on the households’ demand function with respect to the environmental good in question—not the demand schedule for that good. Nevertheless, it is the most reported result in the hedonic literature (Palmquist 2005). However, if a policy brings about a non-marginal change in the environmental amenity in focus, it may likely result in a shift of the hedonic equilibrium due to implied increase in supply, and the hedonic price function, estimated before the change in amenity supply, will not be able to accurately predict the welfare change in the new equilibrium.

However, Bartik (1988) demonstrated that an ex-ante-estimated hedonic price function can be used to predict the welfare change of a non-marginal *localized* amenity change, as this is unlikely to affect the equilibrium in the entire housing market. Too few properties would be affected, which would leave the hedonic price function stable. The interpretation of a non-marginal localized amenity change is therefore similar to a marginal non-localized amenity change, and the ex ante house price function can be used for reliable estimates of the welfare effect of the amenity change.

A final comment here is needed on the fact that the hedonic method by construction can only measure values as perceived by house owners. There may be other users of recreational areas as those implied by OUDS, which obtain a welfare gain or loss. We briefly discuss this aspect below.

#### The Econometric Methods

The functional form of the hedonic house price function is not prescribed by theory. A simple semi-log functional form of the hedonic price function is chosen based on the findings of Cropper and others (1988). Other functional forms were investigated and largely resulted in the same patterns.

The house price function was estimated using four different models. One was a simple non-spatial OLS estimation whereas the three other models contained a spatial autoregressive error term which corrects for the presence of spatial autocorrelation. Due to problems of endogeneity, the spatial models are estimated using maximum likelihood (ML) and the GMM estimator (Kelejian and Prucha 2010).

The spatial econometric model follows Anselin’s (2010) original definition of the spatial error model. It includes a spatial autoregressive error term which corrects for spatial autocorrelation. The specific spatial error model that we arrived at and applied in the valuation can be written as follows:

$$ \begin{aligned} \log (y_{n} ) = & Z_{1n} \beta_{1} + r_{{{\text{access}},n}} \beta_{2} + r_{{{\text{size}},n}} \beta_{3} + \left( {\frac{1}{{r_{{{\text{negative}},n}} }}} \right)^{2} \beta_{5} + \log ({\text{lake}}_{n} )\beta_{6} + \varepsilon_{nm} \\ \varepsilon_{nn} = & \lambda W\varepsilon_{nm} + u_{n} \\ \end{aligned} $$

Here *y* is the price of the *n*’th house, which is a function of the vector *Z* consisting of several structural, neighborhood, and environmental variables not in focus here. Several variables and transformations of these were evaluated to find a set that performed well and enabled us to capture the benefits of various types of green areas and the presence of water in these.

It was found that the group of green areas that contained features such as lakes and trees could be aggregated into one. The impacts of proximity to these green areas as well as the impact of their size were captured in the hedonic price function with the proximity to the nearest green area measured in beeline distance *r*_access_ (in 100 m) and size measured in hectares.

A second group of urban green spaces was identified as areas without trees or lakes, i.e., typically open grass areas with no other features. The impact of these on the price of nearby properties was captured using the measure, *r*_negative_, which is the beeline distance to the nearest such urban green space areas. It was found that a transformation of this distance as a squared inverse provided the best model fit. This transformation depicts a sharp decline in spatial effect. Only the very close neighbors were affected by this second group of green spaces. The inverse distance is also used in other studies, e.g., Anthon and others (2005). In addition, the model contained a term which describes the value of proximity and access to lakes, lake_*n*_. This accessibility measure was defined by the natural log to the beeline distance to the nearest lake.

Finally, we allowed for spatial autocorrelation in the error term *ε*. *W* is an *M* × *M* spatial weight matrix of autocorrelation in errors and *u* is assumed i.i.d. The spatial weight matrix *W* defines the extent of the spatial neighborhood effect at each location. The spatial autoregressive error term in the spatial error model can be understood as a correction term for omitted variables, which are shared by the local neighborhood.

## Case Study

### Area Description

**Fig. 2:**
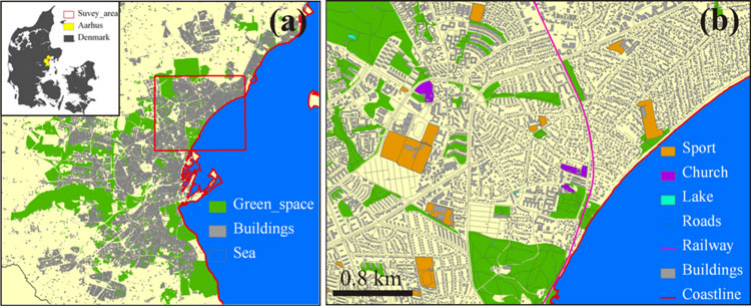
Location of the survey area of Risskov (**a**) and a close overview of Risskov (**b**)

The analysis covered two survey areas: an area for CBA analysis of climate adaptations and an area for estimating the hedonic price function applied in the CBA. The CBA area is restricted to the urban catchment of Risskov located in the northern part of the center of Aarhus city (see Fig. 2). Risskov is one of the wealthiest residential areas in Aarhus with high property values. The catchment size is about 377.3 ha. Commercial and industrial activities are marginal in the area. Risskov has several large green spaces and therefore has a great potential for decentralized drainage constructions. The mean annual precipitation is about 650 mm in Risskov and the highest elevation is 70 m above sea level. A separate sewer system conveys storm water from west to the outlets along the eastern coastline. The region has experienced a few precipitation extremes in recent years, e.g., the extreme rain event on May 3, 2005 with around 50 mm rain in 140 min, and the event on August 1, 2006 with around 56.2 mm in 266 min.

The area that formed the basis for estimating the hedonic price function covered the entire city of Aarhus. The location of the green spaces is shown in Fig. 2. It is seen that green space is widespread throughout the city of Aarhus. Less than 25 % of all properties in Aarhus are located more than 500 m from the nearest green space. The size of green spaces included in the valuation varies between 1 and 741 ha with a mean of 9.5 ha and a standard deviation of 48 ha. Furthermore, the hedonic valuation involves 12,339 properties sold between 2000 and April 2010. Apartments are not considered in this study as only few apartments within the area would be affected by the location of OUDS and/or new green spaces. In addition, we consider that apartments are a separate housing market, and it would lead to bias if we included them in this analysis.

Due to the long planning horizon in this study, potential changes in city environment (e.g., population growth, socio-economic development) are important to include in the analysis. However, the residential catchment is relatively small and well developed and it is, therefore, assumed that there will be no dramatic changes in the city environment in the foreseeable future, see Table 1.

**Table 1:** Assumptions applied in the cross-disciplinary analysis

Subject	Assumptions	Reasons for assumptions
*Climatic loadings*		
Rainfall input	Chicago design storms (CDS) are used as rainfall input Rainfall is homogeneous over the catchment	The CDS provides sound information for urban drainage design because antecedent conditions are not important for the design loading of urban drainage (e.g., Butler and Davies 2011). Advantages are computational efficiency and non-biased estimates of precipitation loading
Climate change impacts	Climate factors (CF) are applied to describe climate change impacts on precipitations over time	Current Danish drainage design guideline uses CF to dimension future rainfall events (Arnbjerg-Nielsen 2012).
*Hazard simulation*		
1D and 2D coupled inundation modeling	Runoff from paved areas is collected through subcatchments in the 1D sewer model. As runoff increases, water can flow out to the 2D terrain model through couplings once the drainage gets overloaded. The couplings allow for water exchange in both directions between the two models	A “compromise” solution to achieve relatively accurate representations of overland flow paths and dynamics with less demanding data and computational requirements (Domingo and others 2010; Mark and others 2004; Timbe and Willems 2004; Zhou and others 2012). Further, a better description of the overland flow dynamics requires a better process understanding and better data than what is available today
Imperviousness	Imperviousness is defined as a subcatchment parameter in the 1D model. The 2D terrain model is impervious	
*Vulnerability assessment*		
Stage-damage function	The threshold principle adopts a binary approach: “flooded or not flooded” depending on whether the inundation depth exceeds the threshold or not. Uniform unit costs are assigned to flooded units	A lack of sufficient information on a credible regional staged-depth-damage function due to urban complexity (Kubal and others 2009; Zhou and others 2012)
Future changes	There will be little change in the city layout and socio-economic conditions. City development and population growth are not considered in this case study	Future changes can be expected due to the long planning horizon; however, it is difficult to tell whether the city will be more vulnerable or resilient The catchment is relatively small, well developed and has not changed much over the last decades. The main land use is residential and changes are not foreseen in planning documents
*Risk reduction*		
Design criterion	Combination of two types of decision criteria: D1: Uniform service level (5-year) based on the equity principle D2: Economically optimal approach considering both costs and benefits	Depending on topographical and land use conditions, adaptation based on D1, in some cases, may be very costly and thus lead to uneconomical solutions. D2 is used to supplement D1 in such cases (Zhou and others 2012)
*Adaptation alternatives*		
Model setup of infiltration	The runoffs are directly removed from the selected subcatchments by reducing the imperviousness. Infiltration capacity with regard to rainfall depths and duration, soil condition is assumed to be constant over the entire catchment and sufficiently high to avoid spilling from local infiltration units to the drainage system/overland flow	Infiltration process is simplified due to a lack of data and advanced models for infiltration simulation
Technical feasibility of infiltration	Actual context of the catchment (e.g., available space, system maintenance, ground water level) is not taken into account	A lack of information and data on the local conditions of the catchment
Model setup of OUDS	OUDS strategy is performed by creating local depressions and flow paths in the DEM. The depressions are impervious	A simplified way to achieve reasonable representations of OUDS
Technical and recreational feasibility of OUDS	The geographic restrictions and legislation limitations of OUDS are not considered. The OUDS have negligible volumes of water at the time of large storms implying that the entire volume is used to minimize floods while still containing sufficient volume on a day-to-day basis to provide the services provided by the natural systems used in the hedonic price analysis	It is beyond the scope of this paper to assess fully the complex dynamics of the OUDS, which would include a detailed ecological and hydrological model of the systems. It is recognized that such an analysis most likely would lead to the OUDS requiring more space and/or provide less services than the actual natural systems. As such, the calculated benefits may be an optimistic estimate compared to the other adaptation alternatives
*Costs and benefits*		
Pipe	Full construction costs are used for assessing investment costs rather than the marginal costs of using larger pipes	Some studies use the marginal costs of using larger pipes assuming that regular operation and maintenance will cover the cost of regular replacement. However, since the pipes are replaced in the beginning of the planning period, the synergy with regular operation and maintenance is negligible
Infiltration	Both the infiltration units and OUDS are renewed every 30 years, thus, in total three investments are needed	The technical lifetimes of infiltration units and OUDS are in general short (Achleitner and others 2007; Bergman and others 2011; Nascimento and others 1999)
OUDS		
*Hedonic valuation*		
Baseline scenario	The hedonic valuation estimates of marginal values of additional green–blue spaces can be validly used to assess the value of additional space used for OUDS for the surrounding neighborhood	This is related to the scale of our scenario, which will induce a change in environmental amenities clearly marginal in relation to the overall supply of such areas in the housing market area underlying the hedonic function
Evaluation scope	The hedonic method only accounts for costs and benefits reflected in how property prices and therefore also property taxes change with changes in e.g., environmental variables. Thus, it is only an approximation of the possible social and environmental benefits In this study, the additional benefits refer to the increase in property values and taxes due to the recreational design of the OUDS system	The major economic benefit from the OUDS design at the neighborhood level is the increased property values and the resulting increase in property taxes. The hedonic method can capture at least a substantial part of such additional values

### Rainfall Input and Socio-Economic Data for Flooding Loss

When analyzing runoff from individual rainfall events, the internal spatial and temporal characteristics of precipitation have a large impact on the maximum discharges and antecedent conditions may also be important (Arnbjerg-Nielsen and Harremoës 1996; Segond and others 2007). Therefore, the modeling software used to calculate the inundations accepts rainfall input with high spatio-temporal resolution. However, when assessing the average properties of runoff from precipitation extremes from urban catchments simple point estimates of intensity–duration–frequency remains a state-of-the-art approach as indicated by e.g., Arnbjerg-Nielsen and Harremoës (1996) and Willems and others (2012). The description adopted is, therefore, to use Chicago design storms (CDS) as input rainfall to urban inundation modeling. It is a synthetic rain event constructed to represent a loading of sewer system that corresponds to a prescribed return period for the entire urban catchment. The CDS is estimated based on regional intensity–duration–frequency relationships with inputs of rainfall variables, such as the mean annual precipitation, rainfall location and duration, and return period (Madsen and others 2009). The key assumption of using CDS is that antecedent conditions of the catchment play a minor role in the calculated extend of the floods for extreme precipitation, see Table 1. We have applied CDS rainfall of return periods of 2, 10, 50, and 100 years for hazard map simulation.

The expected increase in precipitation extremes due to climate change and the associated uncertainties have been studied extensively recently as reported by e.g., Arnbjerg-Nielsen (2012), Larsen and others (2009) and Madsen and others (2009). The current Danish urban drainage design practice suggests a 20, 30, and 40 % increase for the 2-, 10-, and 100-year frequency, respectively, over a 100-year planning horizon. These values are, therefore, used to assess the impacts of climate change in this study. This means that the estimated flood magnitude and frequency of the present return periods will increase in future. For instance, the investigated 100-year event will become a 20-year event after 100 years. As a result, a significant increase in flood risk is expected due to climate change.

The DEM used for inundation modeling is derived from LIDAR data and has a grid resolution of 2 m with a root mean square error of the elevation below 0.05 m. Socio-economic data (e.g., unit costs) together with applied threshold criteria for flood damage estimation are derived from regional databases on climate adaptation studies, documented by Zhou and others (2012).

### Strategies for Future Drainage Design

The four adaptation strategies considered relevant to the catchment are described in the following subsections, including their assumptions and restrictions. Two types of decision criteria are applied, see Table 1. Decision criterion 1 proposes a uniform service level corresponding to no surcharge at the current 5-year event. This design criterion is prioritized in the case study to achieve an acceptable risk level of flooding in the area. However, in some cases, adaptation based on Decision criterion 1 may lead to very costly and uneconomical solutions because the adaptation strategy is not very well suited to solve the problem in particular parts of the catchment. In such cases, Decision criterion 2, the economically optimal approach, is applied to insure an efficient allocation of investment by weighing both costs and benefits. This means that although, for some of the areas, the minimum service level is not fulfilled, the actual flood damage is expected to be at a level acceptable to society. For a given catchment, critical areas with overloaded manholes are first identified based on inundation modeling. Adaptation measures are subsequently applied to the areas to comply with the service level. Meanwhile, the efficiency of the proposed measures is evaluated to assess the corresponding costs and benefits. Decision criterion 2 is adopted in case the proposed adaptation is not economically beneficial. As a result, the proposed measures for each strategy have been assessed based on a manual heuristic trial and error approach which optimizes the efficiency in terms of risk reduction.

#### The Laissez-Faire Strategy: Climate Change Impacts in Risskov

The laissez-faire strategy exposes a situation where no adaptation activity is initiated to cope with climate change impacts. Such a strategy may lead to increased costs of flooding in the future. In this study, it serves as a baseline for evaluating the efficiency of other proposed adaptation scenarios.

#### BAU: Pipe Enlargement

**Fig. 3:**
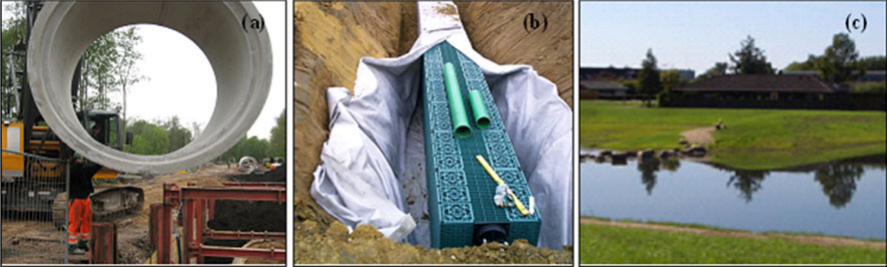
Examples of three of the adaptation options: **a** Pipe enlargement, **b** infiltration trenches, and **c** recreational basins (Arkil 2012; CCA 2012)

Conventional handling of climate change impacts is based on a series of sewer solutions, including optimization of transport capacity of existing sewers, implementing additional pipes or storage spaces, increasing existing pipe capacity, and so on. We applied pipe enlargement in this study as the BAU scenario to enhance existing sewer capacity for excess flows. This is done by replacing relevant pipelines with larger pipes, see Fig. 3a. The implementation of such a solution in inundation modeling is performed by increasing the pipe diameter of relevant links in the 1D sewer model.

Note that the pipe enlargement solution may potentially have minor impacts on received water quality since the increased urban runoffs contain more pollutants from roofs and roads. Additional end-of-pipe solutions may be needed to improve the water quality. The enlargement process may also influence the local traffic conditions including causing traffic inconveniences, road renovation, etc. However, it is difficult to take all of these impacts into account. In this study, we only assessed the direct impacts in the hydrological context.

#### Local Infiltration

This scenario is aimed for infiltration with water trenches, which has been increasingly sought and promoted in the literature and demonstration projects (Wong and Eadie 2000; Stahre 2006). The solution has effects on slowing down and attenuating water flows; however, it may have very limited effects on extreme rain events in some regions due to geological and spatial limitations. As shown in Fig. 3b, local infiltrations were implemented in the form of infiltration trenches, with green coverings (e.g., grass, vegetation) on the top of the sub-surface devices. However, such details cannot yet be modeled with the available program and models; a simplified approach (Table 1) is thus applied by reducing the imperviousness of selected sub-catchments included in the runoff component in the 1D model as a representation of disconnections of subcatchments and water infiltrated into the ground.

We assume that there is no additional effect due to this approach even though concerns have been raised on the rise of ground water which in a worst case assessment could cause widespread basement flooding and structural instability of many tangible assets (Roldin and others 2012). Water contamination from urban pollution has also been raised as a serious issue which ultimately could result in contamination of ground water and drinking water (Birch and others 2011). Furthermore, from a welfare economic point of view, there is no additional recreational benefits from local infiltrations. This is due to the assumption that all infiltration trenches are implemented as invisible structures under existing green spaces (gardens) in Risskov. As a result, no marginal changes/benefits can be observed by local neighborhoods.

#### Open Urban Drainage System (OUDS)

Green spaces in the urban landscape provide amenity services to the surrounding neighborhood in the form of recreational opportunities. The concept of OUDS implies that such a facility is concealed as green recreational sites which are designed to have the additional function of serving as a temporary detention sink for precipitation. Such a solution can exploit new aspects for urban drainage design on recreational amenities, multiple uses, see Fig. 3c. As a result, the economic performance in terms of cost-recovery may occur at different stages of the planning process compared with the conventional solutions.

In the modeling, the OUDS solutions are constructed by creating local depressions/holes in the existing DEM to represent the basin location and size, see Table 1. The potential locations of the green features are first identified based on the inundation modeling. It can be noted that the OUDS solutions are mainly located on the pathway toward or directly in the potential flooding zones. The efficiencies of the proposed locations are subsequently evaluated using the flood risk assessment and economic analysis to estimate their net benefits. Priority is given to locations with higher benefits. In doing so, it is possible to achieve a reasonable optimization of OUDS locations based on a trial and error approach. Furthermore, the scenario was implemented with two subcategories in the model. This is because to attain good performances on flood mitigation some OUDS need to be located in private gardens or spaces. Such OUDS will mainly perform as rainwater basins in the area while OUDS located in green spaces are assumed to be designed as a lake integrated in urban landscape. These two settings differ from a socio-environmental point of view and will lead to different impacts on the economic assessment.

The feasibility of achieving both the technical functionality of OUDS and the amenity value is not considered in this study, see Table 1. Such systems are studied in many regions in the world and it remains an issue to insure that the systems can in fact perform as well as natural systems in terms of continuous provision of positive environmental values and functions. Typical problems are drying out, eutrophication, overgrowing, and/or heavy maintenance requirements. The costs of, e.g., maintaining nutrient balances by removal of excessive plant growth, installing and maintaining systems that may artificially add water in dry periods, are not included in the cost-benefit analyses presented.

### Assumptions and Simplifications in the Study

Due to the complexity of the cross-disciplinary approach, several important assumptions were made to simplify the integrated analysis in this study, as summarized in Table 1. Overall, the assumptions seem reasonable, and indeed necessary to reach an evaluation of each of the strategies. Based on Table 1, it may appear that the benefits of the OUDS systems may be exaggerated somewhat, which should be taken into consideration when comparing strategies. Since it is not possible to quantify the importance of this potential exaggeration in economic terms, we will discuss the importance in qualitative terms as part of the discussion and conclusion of the paper.

## Results

### Flood Risk Assessment

**Fig. 4:**
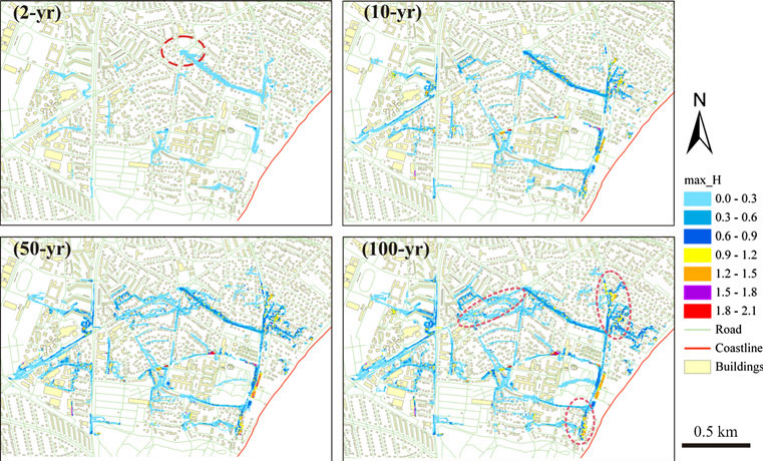
Simulation of inundated extent and depth using Mike Urban and Mike Flood software package

The flood hazard maps indicating the current hazards are shown in Fig. 4. The calculated depths are the maximum water depths observed for each of the recurrence intervals indicated in the figure. A 5 × 5 m grid was applied for surface flood modeling to achieve a balance of computing time and accuracy. There is a severe overloading of the sewer system near the outlet in the north center, as marked in the hazard map of the 2-year event. Several local flood-prone areas were identified from the maps, as indicated in the hazard maps of the 100-year recurrence interval.

To calculate the damage costs for individual rainfall event, the hazard maps were incorporated with the land use map in GIS to give a visualized overview of the potential damages in the area. We assessed the number of flooded properties for each damage category on the basis of the GIS-map and estimated the total costs as a summary of the individual costs of each damage category. The unit costs used for assessing the costs are identical to the ones used by Zhou and Arnbjerg-Nielsen (submitted).

### Hedonic Valuation

The hedonic price functions included a large number of control variables that cover structural, neighborhood, and environmental characteristics of the property. Each property is geo-coded with its exact location which enabled very accurate location-based variables describing neighborhood and environmental characteristics of each property’s surroundings. Data on property sales and structural characteristics of the property were obtained from the OIS database (Hansen and Skov-Petersen 2000). The location-based variables were constructed using GRASS (6.4) (2008) and ArcGIS (9.3). The GIS data are provided by the National Survey and Cadastra. The model was estimated in R while using the spdep package and the sphet package (Bivand and others 2011; Gianfranco 2010; Team 2011).

The parameter estimates of the variables are robust in terms of size (within same order of scale) and significance over three models, differing in their modeling of the error term only, see Table 2. The significance levels of the variables vary slightly between the models. The OLS model resulted in highly significant parameter estimates for all parameters of interest. The robust spatial error model has less significant variables with the log(lake) variable only being significant at the 10 % level. The non-robust error model performs the poorest with log(lake) being non-significant and *r*_negative_ being significant only at 10 % level. The spatial variables in the OLS model are likely to capture some of the spatial autocorrelation which is not related to the variable itself, and hence, the parameters may suffer from an omitted variable bias due to the assumption of an i.i.d. error term. It seems that especially *r*_negative_ and log(lake) are sensitive to spatial autocorrelation, being both more significant and having larger parameter values (though not significantly) than the estimation results of the spatial error models revealed.

**Table 2:** Model results of the hedonic price function

Variable	OLS	ML-ERROR	Robust GMM-ERROR
*r*_access_ (100 m)	−0.005432*** (0.001357)	−0.00709* (0.00296)	−0.00688** (0.002748)
r_size_ (Ha)	0.000112*** (0.000021)	0.000112*** (0.0000339)	0.000113*** (0.000036)
*r*_negative_ (100 m^−2^)	−2.095384*** (0.623251)	−1.1321** (0.26)	−1.1809*** (0.434954)
Log(lake) (100 m)	−0.036296*** (0.005006)	−0.01433 (0.01009)	−0.01722^†^ (0.009944)
λ		0.72512*** (0.01409)	0.7457*** (0.02344)
*N*	12,339	12,339	12,339
*R* ^2^ (pseudo-Efron)	0.63	0.68	0.63
Breush–Pagan test	4092.657***	1663.095***	
Log likelihood	−2143.153	−1332.092	
AIC	4356.307	2736.2	

^†^ Significant at 10 %

* Significant at 5 %

** Significant at 1 %

*** Significant at 0.1 %

Due to these observations, we decided to apply the results of the Robust GMM model with spatial autocorrelation accounted for.

We used a row standardized 30th nearest neighbor weight matrix, *W*, in the spatial error models, which proved sufficient to account for the autocorrelation revealed by global and local Moran I tests on the residuals of the simple OLS model, as well as spatial correlogram analysis (Cliff and Ord 1981). The Lagrange Multiplier test for spatial error dependency and spatial lag dependency are both highly significant (Anselin 1988). The robust version of the test indicates that the spatial error model outperforms the spatial lag model. Heteroscedasticity is a problem in the OLS model and the spatial error model based on ML. The robust spatial error model based on GMM provides the most trustworthy results.

The dependent variable in the models was the natural log to the house price. Thus, in the robust GMM model, we found that the marginal value of accessibility to the urban green areas, which included lakes or tree cover or both, decreased with 0.6 % of the property price for every 100 meters a house was removed from such an area. The marginal value of an increase in the size of the nearest such urban green area was 0.01 % of the house price for every additional hectare. The urban green areas not including lakes or tree cover affected the very nearby properties negatively, as seen on the parameter for *r*_negative_. On the other hand, access to nearby lakes, including those not integrated in a green area, was exponentially related to the house price which means that a 1 % increase in distance to a lake will reduce the property value with 1.7 %.

While the parameters all have the expected sign and are significant, it should be stressed that the effects they imply are in fact quite small compared with that of, e.g., proximity to forests and similar effects often found in other hedonic studies (e.g., Anthon and others 2005). Nevertheless, because of the high aggregate value of the properties in the areas, the effects of enhanced environmental amenities may still be significant.

### Integrated CBA

#### Laissez-Faire Strategy

Owing to climate change, the EAD was estimated to increase from 8.3 to 17.8 MDKK (10^6 ^Danish Kroner) from year 2011 to 2100 if discounting was ignored. That is to say, the total added damage costs due to anticipated climate change will be 92.7 MDKK in the present form if no adaptation is planned for the area. This value can be considered as an indication of the levels of investment allowed for adaptation from a cost-benefit point of view. In addition, it is noteworthy that the estimated value only reflects the expected damage on an average level, and the real costs may be several times higher in the worst case. Early actions can be recommended to tackle the climate change.

#### Pipe Enlargement

**Fig. 5:**
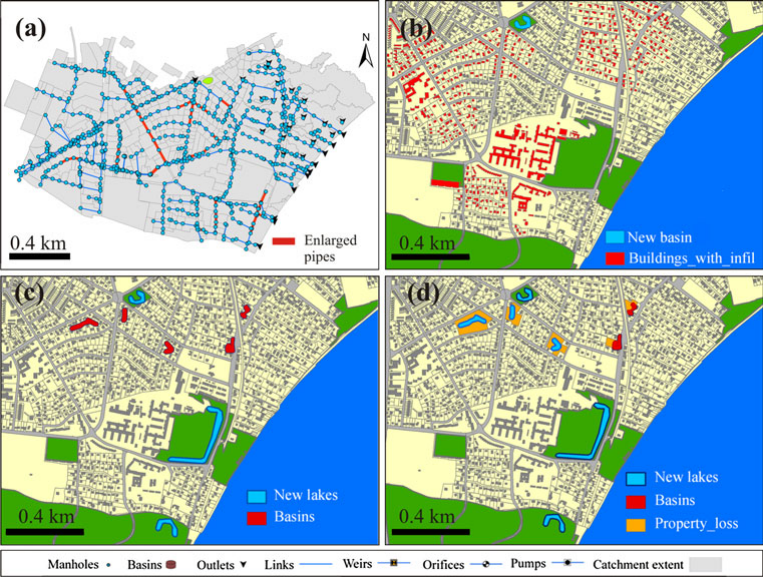
Illustration of **a** pipe enlargement, **b** infiltration, **c** OUDS 1, and **d** OUDS 2. The figures show where measures are suggested for tested adaptation scenarios based on an optimization of costs and benefits

To achieve an acceptable risk reduction by pipe enlargement, in total 2636 meters of pipe need to be enlarged, see Fig. 5. The investment unit costs will increase as a function of pipe diameter with 7,000 DKK/m as an average estimate. The total investment costs for pipe enlargement were calculated to be 24.1 MDKK. It is a one-time payment invested evenly in the first five years of the planning horizon. Moreover, it can be noted that there is an extra open basin invested for both pipe enlargement and infiltration. This is because the extra water from the overloaded sewer in the north center (Fig. 4, 2-year event) requires intensive adaptation measures in the area if handled by pipe enlargement and infiltration individually.

With pipe enlargement, the original EAD in 2100 was reduced from 17.8 to 8.4 MDKK per year. The calculated net benefits of the solution are 147 MDKK over a 100-year planning horizon. Nevertheless, it is noteworthy that adaptation by pipe enlargement needs dramatic changes in the sewer system if lower flood risks are to be achieved.

#### Infiltration

It is estimated that a large part of Risskov will need to be disconnected from the sewerage system when applying this strategy. In total 14.53 ha impervious area had to be disconnected, corresponding to the roof area of 727 buildings. The areas to be disconnected should be upstream of the inundated areas to be effective in minimizing the flood hazard. The locations of these buildings are shown in Fig. 5. The unit cost for implementing the infiltration trench is 250 DKK/m^2^ plus 60,000 DKK per property owner. This estimate is based on empirical data from the facility company Bornholm Vand and A/S which in cooperation with local citizens decoupled several streets in the town of Allinge.

Using this strategy, the calculated total investment costs are 87.1 MDKK accounting for two reinvestments later to consider the low technical life time of infiltration devices. The estimated net benefits are around 111 MDKK. However, we want to address that the implemented infiltration is an optimistic scenario in the case without considering restraints due to low permeability soils and high ground water levels in the area. The hydrological response process of infiltration is also simplified. The practical performance may have much lower efficiency in reducing the hydrological loadings to sewer system.

#### Open Urban Drainage System (OUDS)

Based on the model simulation, 49,558 m^3^ of storage volume will be required in the strategy, for detailed information see Fig. 5. Unit costs of 745 DKK/m^3^ are used for estimation of investment costs (PH-Consult 2006). We divided the OUDS strategy into two subscenarios: OUDS 1 and OUDS 2. In the OUDS 1 scenario, we assume that basins located on private properties will take up parts of the garden of the property. In this scenario, three lakes are located within existing green spaces. Two of the green spaces initially without lakes or tree covers had negative impacts on nearby properties. However, in this scenario, their category is changed from being a negative green space to a positive green space in the hedonic valuation after obtaining the lakes. In addition, the five rainwater basins located on private properties took up garden space. In total, 35 properties lose parts of theirs garden. In the OUDS 2 scenario, we assume that properties affected by rainwater basins in OUDS 1 are converted into green spaces with smaller permanent lakes. Two of the affected areas are too small to be considered as green spaces and will, therefore, still be categorized as rainwater basins, which was found to have no hedonic effects. In total, six new small lakes and three new positive green spaces are located within the survey area. In total, 35 single family houses are removed along with their entire property.

In the context of flood reduction, the EAD decreased to 6.3 MDKK per year with implementation costs of 54.5 MDKK in the present value. The estimated net benefits from the conventional CBA are 157 MDKK.

The welfare changes of the two OUDS scenarios were further calculated based on results of the robust spatial error model of the hedonic price function. The welfare estimates used properties from all of Aarhus municipality (see Table 3). OUDS 1 provides a potential welfare increase of 223.1 MDKK and OUDS 2 provides a potential welfare increase of 154.0 MDKK, which account for 1.48 and 1.03 % increase in value of affected properties, respectively. In total, 3,450 properties would be affected by the changes in OUDS 1 and OUDS 2. The scale of the change in the urban landscape and the expected welfare change are of a magnitude that can be considered localized in relation to the overall Aarhus housing market, assuming the OUDS solutions are only implemented in Risskov. Thus, the estimated welfare changes of the OUDS should be considered an upper bound measure not likely to be valid as a central estimate if OUDS are applied widespread in the city. On the other hand, the hedonic method only includes the benefit of these areas as experienced by the local home owners affected directly. There may, in some cases, be effects also for people further away. In the present case, however, the spatial extent of the green areas established are small compared to the overall supply of larger green recreational areas in and around Aarhus.

**Table 3:** The average benefit in MDKK (million DKK) from OUDS 1 and OUDS 2 based on welfare estimated from the estimated hedonic price function using the robust spatial error model

Source of welfare economic loss/gain	OUDS 1	OUDS 2
Loss of property		−179.61
Loss of garden	−22.28	
Increased access to lakes	86.11	144.63
Loss of unattractive recreational area	0.22	0.22
Increased access to recreational areas with lake	141.39	169.87
Increase in size of nearest recreational area	17.64	18.86
*A: Sum of direct increase in property prices*	*223.09*	*153.97*
Total property value of affected properties	15071.60	14960.48
*B: Tax revenue*	*177.16*	*122.23*
Total	400.25	276.24

The numbers in italics show the added economic benefits due to increased property values in the area and the resulting increase in property taxes

The environmental amenity changes in the two scenarios would be capitalized in the property market if implemented. In Denmark, part of the property tax is collected as a percentage of the property value. In this situation, part of the resulting welfare change will not be reflected in the house price change, but instead in increasing property taxes acquired by the taxation authorities. Thus, not accounting for property tax will underestimate the true welfare change (Anthon and others 2005). In Aarhus municipality, the property tax is 2.458 % of the property value. The additional value acquired by the municipality over a 100-year period with a discount rate of 3 % will sum to 177 MDKK for OUDS 1 and 122 MDKK for OUDS 2.

### Summary

The estimated cost reductions in investigated rainfall events and EAD under climate change impacts are summarized in Table 4. The calculated NPVs of the four strategies based on the traditional CBA and extended CBA including hedonic estimation are shown in Table 4 as well. It was found that all investigated adaption strategies are economically beneficial relative to the *laissez*-*faire* alternative. The largest gain was found for the OUDS solutions in this area, and there is a considerable increase in estimated NPV when taking into account the additional environmental amenity benefits that the OUDS imply. Note that this happens in spite of relatively small, but significant increases in property prices that will occur from, e.g., establishing a new urban green area with a lake or improve existing areas with lakes.

**Table 4:** Estimated cost reductions in investigated rainfall events and expected annual damages assuming climate change (CC), as well as total investment costs demanded for the four strategies

	Return periods				EAD with CC	Infrastructure investment costs	NPV1	NPV2
	2-year	10-year	50-year	100-year				
(MDKK)								
Laissez-faire	7.29	18.09	35.69	43.28	17.8	0	−93	−93
Pipe enlargement	3.98	7.63	19.46	23.55	8.37	24.07	147	147
Local infiltration	1.21	4.12	12.62	18.80	4.63	87.12*	111	111
OUDS 1	2.42	6.04	15.46	22.47	6.25	54.50*	157	557
OUDS 2	2.42	6.04	15.46	22.47	6.25	54.50*	157	433

Note that the investment costs are calculated in NPV with a discount rate of 3 % for a 100-year horizon. The NPV1 and NPV2 denote the calculated net benefits from the conventional and extended CBA, respectively

* Three investments were assumed needed over the planning horizon

## Discussion

This study compares a *laissez*-*faire* strategy of inaction, a traditional business-as-usual enlarged drainage solution, local infiltration solutions, and OUDSs for climate change adaptation. The results indicated the conventional drainage solution (e.g., pipe enlargement) was cost efficient in terms of flood risk reduction, however, incapable of integrating other positive perspectives in the drainage facilities, such as amenity values. Rebuilding the pipe system may be relevant to areas where small-scale renovation is required to improve the runoff conditions, or areas where no open space is available for decentralized solutions. Our results were more supportive of OUDS, which can be considered as a significant supplement or replacement of the traditional solutions owing to its positive impacts on recreational and environmental aspects in urban context. Especially under the influences of climate change and city development impacts, such approaches may prevail over the traditional solutions since OUDS can be better integrated in urban landscape for excess surface waters as well as strengthen the efficiency of multiple land use. Certainly, we also stress that the open drainage solution may not be as relevant and beneficial for areas where access to amenities and water is already widespread (and the marginal value of more amenities is therefore low), or in areas where costs (in terms of land e.g.,) of space for such a system are much higher than traditional solutions. In some cases, due to technical reasons (e.g., pollution control, safety issues, and legal constraints), open drainage solution may not be the appropriate way of adaptation either. However, it may very well be that in many cases, OUDS has the capacity to integrate different recreational activities in the drainage facilities, which is especially relevant to areas with a lack of blue–green features in a large-scale neighborhood or areas where multifunctional drainage solutions are required. The assumptions behind the analysis are likely to favor the OUDS solutions, in the sense that probably the systems will require more space to provide as much value as natural systems (increasing space requirements and land and construction costs) or alternatively be less attractive than the areas the estimates are based on and hence yield less value to the neighborhood (reducing the welfare benefit). However, the numbers are quite unambiguous in the sense that even without taking the recreational gain into account, the OUDS systems are attractive from an economic point of view as a means of flood risk mitigation, and, even without considering the flood risk mitigation, the OUDS systems are economically attractive because of the welfare gain from amenity values.

The uncertainties involved in the methods presented in this study are substantial. The 1D–2D coupled model is a “compromise” modeling approach to achieve relatively accurate representations of overland flow dynamics with reasonable—yet extensive—amounts of data and computational requirements. Such an approach involves uncertainties associated with input data, system setup, model parameters, and assumptions (Domingo and others 2010; Freni and others 2010; Koivumaki and others 2010; Timbe and Willems 2004). The setup of the applied adaptation options has been simplified in terms of both modeling simulation and economic assessment as discussed in Table 1. Nevertheless, the results seem unequivocal in the sense that the differences in net present value between the analyzed strategies are substantial.

The results highlight the difficulties in setting up the proper framework for the analysis and how the results should be interpreted. A traditional framing approach would be to consider only the urban drainage sector in the analysis, leading to the result that pipe enlargements and open basins are equally suitable as adaptation measures against increased risk of flooding.

When framing the analysis to include potential benefits of the OUDS; however, this solution turns out to be very likely best solution of the options considered. However, the value of the added recreational benefits is estimated under the assumption that only this part of the city will implement OUDS, and hence the change in environmental amenities is marginal in relation to the overall housing market captured in the hedonic function. If the entire city chooses to implement OUDS, the benefits are likely to be smaller than those estimated here, and the estimates should, therefore, also for this reason be considered an upper bound. This is because a widespread implementation of OUDS may affect the housing market’s marginal pricing of the environmental benefits offered by OUDS, as supply change is no longer marginal. Thus, caution should be taken if one wishes to upscale the results presented here.

Other environmental costs arising from the different scenarios have not been considered in the present analyses, and little actual information is available that can be linked to the adaptation scenarios presented. The amount of pollutants present in the different fractions of water will vary between the scenarios, will have very different fates across the proposed scenarios, and hence, will present different threats to ground water quality, environmental status of recipients, etc. European legislation tends to put high emphasis on surface water, which would tend to favor infiltration and OUDS. However, Danish legislation puts high emphasis on ground water protection, which would tend to favor traditional sewerage expansion. Thus, adding these additional environmental concerns is likely to draw conclusions in different directions and complicate the overall choice of adaptation action.

## Conclusions

Our results indicate that there is a large potential for studying and implementing OUDS as a means to both mitigate increased risk of flooding in urban areas as well as enhance the recreational value of local neighborhoods. The results are based on cross-disciplinary methods where risk assessment of urban floods covers the topics of flood inundation modeling, climate change, environmental evaluation tools, and socio-economic tools, to reveal the costs and benefits associated with our four different climate adaptation strategies. A budget oriented socio-economic analysis was found to be a sub-optimal approach for decision making as it will be blind to the potential additional services provided by non-market goods linked with some adaptation scenarios. We find that in the case area, a climate adaption strategy based on OUDS is better than the other strategies, given the framing of the problem, while a strategy of laissez-faire is the least attractive. Our results indicate that the conceptual framework around the decentralized sewerage system needs to be rethought. Retaining the water on individual properties is a more expensive solution than pipe enlargement and does not provide the recreational benefits of open systems with permanent water bodies, which require that neighborhoods have a joint drainage system.

The approach presented in this study is especially suitable for complex evaluations where not only the traditional framing of urban drainage is used, but also a broader perspective is needed. Many studies have dealt with the recreational values of making urban drainage more visible. These studies have discussed the issue in a qualitative manner, but without putting the recreational value on the same monetary scale as traditional engineering methods usually do. This method bridges the gap between the different scales used by engineers, landscape architects, and urban planners and will hopefully, therefore, be a valuable means of choosing between different adaptation options within urban drainage in fully developed cities.

## Footnotes

1 One could expect an effect of these simple rainwater basins on the price of nearby houses, but the hedonic analyses did not indicate such price variations.
